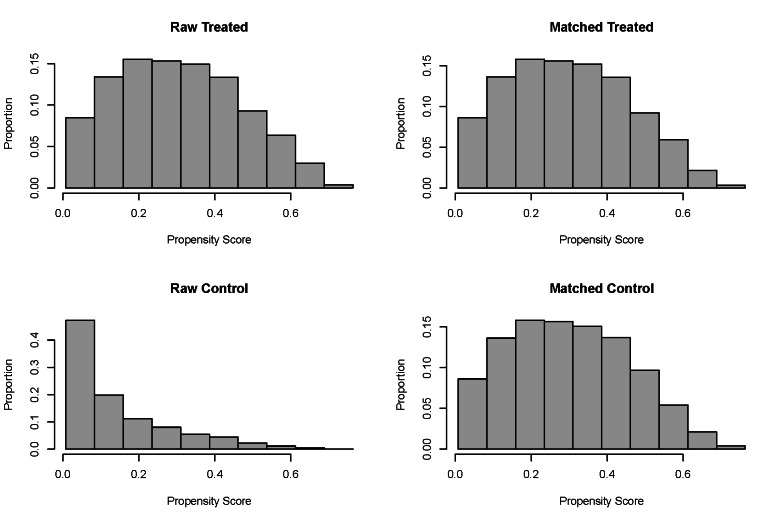# Association between the visceral adiposity index and rheumatoid arthritis: A cross-sectional study based on the NHANES 2007 to 2016

**DOI:** 10.1097/MD.0000000000048744

**Published:** 2026-05-22

**Authors:** Shengcong Guo, Dianbo Yu, Wei Huang, Junnian Yang, Jun Yao

**Affiliations:** a Emergency Medicine Department, The Fourth Affiliated Hospital of Guangxi Medical University, Liuzhou, China; b Bone and Joint Surgery, The First Affiliated Hospital of Guangxi Medical University, Nanning, Guangxi, China; c Guangxi Health Commission Key Laboratory of Clinical Medicine Research on Bone and Joint Degenerative Diseases Cohort, Department of Orthopedics, Affiliated Hospital of Youjiang Medical University for Nationalities, Baise, China; d Emergency Department, Liuzhou Workers’ Hospital Sanjiang Branch, Liuzhou, Guangxi, China.

**Keywords:** NHANES, propensity score matching study, rheumatoid arthritis, VAI, visceral adiposity index

## Abstract

The visceral adiposity index (VAI), a measure of abdominal fat, has shown associations with various diseases. However, no study has investigated the relationship between VAI and rheumatoid arthritis (RA). This cross-sectional study aimed to examine the correlation between RA risk and VAI among US adults. Data from the National Health and Nutrition Examination Survey for the years 2007 to 2016 were downloaded. Participants were categorized into RA and non-RA groups based on arthritis questionnaire responses. VAI was classified both as continuous and in quartiles (Q1–4). All analyses incorporated National Health and Nutrition Examination Survey sampling weights, specifically the fasting subsample 2-year weights (WTSAF2YR), to account for the complex survey design and produce nationally representative estimates. The relationship between VAI and RA risk was assessed through weighted multivariable logistic regression analyses. In addition, a restricted cubic spline analysis assessed the relationship between VAI and RA risk. Subgroup analyses were conducted to investigate associations in specific subgroups. The same analyses were repeated after 1:1 propensity score matching in enrolled participants. A total of 8942 participants were included in the study. Weighted multivariable logistic regression analyses revealed that participants in the highest VAI quartile (Q4) had a 3% increased risk of RA (odds ratio = 1.03, 95% confidence interval = 1.01–1.05) compared with those in the lowest quartile (Q1). The restricted cubic spline curve indicated a nonlinear correlation between continuous VAI and RA risk. Subgroup analysis confirmed that VAI was associated with RA risk in different subgroups. After propensity score matching, only the Q2 VAI group showed a higher RA risk than the Q1 group, with a 7% increased risk (odds ratio = 1.07, 95% confidence interval = 1.01–1.15). Subgroup analysis revealed that the Q2 group had a high RA risk in populations with alcohol consumption, non-hypertension, and non-diabetes. Among US adults, higher VAI levels were associated with an increased risk of RA, particularly in populations with alcohol consumption and without hypertension or diabetes. These findings suggest an association rather than a causal relationship, highlighting the need for prospective studies to further elucidate this relationship.

## 1. Introduction

Rheumatoid arthritis (RA) is an immune-mediated inflammatory disorder that causes joint pain, swelling, and destruction.^[1]^ The mechanisms involved in joint inflammation include abnormal immune responses and cellular immune responses, leading to the infiltration of rheumatoid factor.^[2]^ The 2017 global burden of disease study estimates the prevalence of RA to be 0.27%, with an 8.2% increase in the incidence rate.^[3]^ Although the etiology of RA remains obscure, previous studies implicate smoking, periodontal disease, and occupational exposure as potential risk factors.^[4]^ Simultaneously, obesity is considered a serious public health problem affecting the onset and progression of RA.^[5]^ Leptin, secreted by adipose cells, is directly correlated with the pro-inflammatory phenotype associated with RA. A 2-sample Mendelian randomization study found that the body mass index (BMI) is positively correlated with RA risk.^[6]^ However, BMI may not reflect body fat distribution and visceral fat.^[7]^ The relationship between visceral fat and RA lacks large sample size studies.

The visceral adiposity index (VAI) is a measurement of fat accumulation in the body that utilizes blood lipid and body measurement indices for calculation. Previous studies have found that VAI is closely related to orthopedic disorders. In a study by Sun et al, VAI was independently associated with an increased risk of osteoporosis.^[8]^ Similarly, Chen et al conducted a study and found that VAI is nonlinearly associated with total femur density in adults.^[9]^ In an arthritis-related study, Jiang et al demonstrated that Chinese VAI can predict trabecular bone loss in early RA women.^[10]^ Despite these findings, limited studies exist regarding the relationship between VAI and RA risk, and a large sample study is needed.

Health and nutrition data on the US population are collected through large, stratified, and multistage surveys administered by the National Health and Nutrition Examination Survey (NHANES). The propensity score matching (PSM) method can help minimize the discrepancies in clinical characteristics among groups and has been widely used in large-sample observational research. The objective of this study was to investigate the association between VAI and the risk of RA among US adults using the PSM method from the NHANES database. This investigation has certain clinical and public health relevance, although its implications should be interpreted with caution, given the modest effect sizes observed. First, by employing a comprehensive methodological approach that includes both traditional regression models and PSM, this study enhances the robustness of findings in observational research. Second, the identification of VAI as a potential indicator for RA risk could inform future preventive strategies, particularly given that VAI integrates both anthropometric and metabolic parameters, offering a more comprehensive assessment of adiposity-related health risks than BMI alone. Third, the subgroup analyses provide valuable insights into specific populations that might benefit most from visceral adiposity assessment, thereby contributing to more targeted and personalized approaches in RA risk management.

## 2. Materials and methods

### 2.1. Data extraction and screening

In the NHANES database, 5 modules of data are available. All participants have provided informed consent, and the National Center for Research Ethics Review Committee has approved the research protocol. Each participant is assigned a unique sequence number, facilitating identification across different modules. Furthermore, each checked item is assigned a unique code in NHANES, allowing the combination of data from different cycles to increase the sample size.

The present study utilized data from NHANES cycles spanning from 2007 to 2016: 2007 to 2008, 2009 to 2010, 2011 to 2012, 2013 to 2014, and 2015 to 2016. Demographic data in the module include age, gender, race, education, household income to poverty ratio (PIR), and marital status of participants. The physical examination data module comprises common indices such as waist circumference (WC) and BMI. The laboratory data module encompasses triglyceride (TG) and high-density lipoprotein cholesterol (HDL-C). Serum TG levels were quantitatively determined using enzymatic assays performed on automated biochemistry analyzers. The questionnaire data module holds information on smoking, alcohol consumption, physical activity, hypertension, diabetes, and arthritis. The study inclusion criteria were the following: availability of RA-related data and availability of BMI, TG, WC, and HDL-C data. Exclusion criteria were as follows: information on variables including physical activity, marital status, alcohol consumption, education, and smoking was “missing,” “refused,” or “don’t know”; information on variables including hypertension, diabetes, and arthritis was “missing” and “don’t know”; and information on the PIR was “missing.”

### 2.2. Outcome and exposure variables

The exposure variable in this study was the VAI, calculated separately based on gender. The formula for calculating VAI is as follows:


$$\begin{array}{l} VAI\left(males\right)=\frac{WC}{\left(BMI\times 1.88\right)+39.68}\times \frac{1.31}{HDL-C}\times \frac{TG}{1.03} \end{array}$$

$$\begin{array}{l} VAI\left(\;females\right)=\frac{WC}{\left(BMI\times 1.89\right)+39.58}\times \frac{1.52}{HDL-C}\times \frac{TG}{0.81} \end{array}$$

WC (cm), BMI (kg/m^2^), HDL-C (mg/dL), TG (mg/dL).

The participant had their WC measured horizontally at the iliac crest while standing. They were wearing disposable shirts, pants, and slippers as part of the examination gown. In addition, participants’ weight was measured using a digital weight scale. BMI was then calculated based on the measured weight and height. Blood samples for TGs analysis were collected from participants in the morning, following at least 8.5 to 24 hours of fasting. These samples were obtained through venipuncture at mobile examination centers.

The outcome variable was the participant’s history of RA. The questionnaire data module included medical condition items recorded in the related questionnaire. Participants were required to respond with either “Yes” or “No” to the question, “Doctor ever said you had arthritis?” Those responding “No” were placed in the non-RA group. Otherwise, proceeded to the next question, “Which type of arthritis was it?” Participants responding to “RA” were then assigned to the RA group. A prior study demonstrated substantial consistency (85%) between self-reported and clinically confirmed arthritis.^[11,12]^

### 2.3. Covariates

Apart from the outcome and exposure variables, the remaining variables were considered covariates. Some of these covariates were treated as categorical variables. Race was categorized as non-Hispanic White, other Hispanic, Mexican American, other race – including multi-racial, and non-Hispanic Black. Education attainment was grouped into 3 categories: high school, college or above, and less than high school. Marital status was recoded into 2 groups: married or living with a partner, and living alone (widowed, divorced, never married, and separated). PIR was categorized into lower than 1.5, 1.5 to 3.5, and over 3.5. Smoking status included 3 categories: current smokers, former smokers, and nonsmokers. A current smoker smoked ≥100 cigarettes in their lifetime and is currently smoking, a former smoker smoked ≥100 cigarettes in their lifetime but no longer smokes, and a nonsmoker smoked <100 cigarettes in their lifetime. Physical activity was further categorized into moderate or vigorous physical activity based on the response to the question, “Does your work involve vigorous-intensity activity or moderate-intensity activity?” Alcohol consumption was categorized as yes or no based on the response to the question, “had at least 12 alcoholic drinks/1 yr?” Hypertension and diabetes were classified based on responses to the question, “Ever been told you have high blood pressure/diabetes?” In subgroup analysis, age was divided into 3 groups: 20 to 39, 40 to 59, and 60 years of age or older.

### 2.4. Statistical analysis

Survey methods were employed to analyze complex sampling designs based on strata, primary sampling units, and sampling weights. Given that TG measurements were obtained in the fasting status, the fasting subsample 2-year weights recorded “WTSAF2YR” were utilized for all weight analyses. Categorical variables are presented as the number (percentage) of cases, while continuous variables are represented as means with corresponding 95% confidence intervals. Data analysis involved the application of the chi-square test and the Wilcoxon rank-sum test. In addition, VAI was grouped into quartiles: Q4 (highest), Q3, Q2, and Q1 (lowest).

The relationship between VAI and RA risk was explored using weighted multivariable logistic regression in 3 models. Model 1 did not adjust for covariates; model 2 adjusted for age, race, gender, education, PIR, or marital status; and model 3 adjusted for all covariates. To evaluate the robustness of logistic regression, sensitivity analyses were performed. When VAI was treated as a continuous variable, restricted cubic splines (RCS) were explored to investigate the potential association between continuous VAI and the odds ratio of RA. Subgroup analyses were conducted to identify associations between VAI and RA risk within specific age, gender, smoking, alcohol consumption, hypertension, and diabetes groups. The interaction effect of the VAI-RA risk relation and these subgroup variables was assessed in model 3. To mitigate the impact of confounding variables, a 1:1 PSM analysis with a 0.05 caliper value was performed to adjust for the effects of age, education level, gender, marital status, physical activity status, PIR, race, smoking, hypertension, alcohol consumption, and diabetes. Subsequently, the same method was used for the post-PSM population. Statistical significance was set at two-tailed *P* < .05. Borderline *P* values (.05 ≤ *P* < .10) were interpreted cautiously, considering the effect size, confidence intervals, and consistency across sensitivity analyses rather than relying solely on the *P* value threshold. Data analysis and visualization were conducted using the R software (R Foundation for Statistical Computing), with primary packages including “haven,” “survey,” “gtsummary,” and “matchit.”

## 3. Results

### 3.1. Participant characteristics

The NHANES 2007 to 2016 dataset initially included 50,588 participants. We sequentially excluded 24,996 participants with missing RA data, 14,981 with missing VAI data, and 1669 with missing covariate data. After this screening, 8942 subjects remained for analysis (Fig. S1). Due to the modest effect sizes and exploratory nature of this study, the findings should be considered hypothesis-generating rather than confirmatory. The final cohort included 1546 participants with a self-reported history of RA and 7396 without arthritis. RA participants tended to be older, female, have an education level above high school, have a BMI ≥ 30, have alcohol consumption, and have hypertension. The continuous VAI value was higher in the RA group than in those without (1.65 vs 1.34, *P* < .001). In addition, the proportion of high-level VAI (Q4/Q3) in the RA group was higher than in the non-RA group. Table 1 presents specific characteristics between the 2 groups.

**Table 1:** Baseline characteristics of participants before PSM and after PSM.

Characteristic	Before PSM rheumatoid arthritis			After PSM rheumatoid arthritis		
	No (N = 7396)	Yes (N = 1546)	*P* *	No (N = 1520)	Yes (N = 1520)	*P* *
Age (yr)	41.0 (30.0, 54.0)	58.0 (48.0, 69.0)	<.001	59.0 (48.0, 70.0)	58.0 (48.0, 69.0)	.3
Sex			<.001			.3
Female	3508 (48%)	893 (59%)		831 (56%)	868 (58%)	
Male	3888 (52%)	653 (41%)		689 (44%)	652 (42%)	
Race			<.001			<.001
Non-Hispanic White	3063 (66%)	880 (78%)		745 (74%)	864 (78%)	
Non-Hispanic Black	1409 (11%)	298 (11%)		273 (9.7%)	293 (11%)	
Mexican American	1230 (9.3%)	191 (4.7%)		216 (6.2%)	187 (4.7%)	
Other Race – including Multi-Racial	815 (5.9%)	127 (3.3%)		159 (4.0%)	126 (3.3%)	
Other Hispanic	879 (7.8%)	50 (3.5%)		127 (6.0%)	50 (3.5%)	
Education level			<.001			.6
Above high school	4061 (63%)	679 (51%)		708 (58%)	674 (52%)	
Less than high school	1685 (15%)	488 (23%)		454 (19%)	476 (23%)	
High school	1650 (22%)	379 (26%)		358 (24%)	370 (25%)	
Marital status			.040			.3
Married or living with partner	4460 (64%)	937 (68%)		961 (70%)	925 (68%)	
Living alone	2936 (36%)	609 (32%)		559 (30%)	595 (32%)	
PIR			.600			.4
<1.5	2778 (26%)	593 (27%)		560 (24%)	580 (26%)	
1.5–3.5	2344 (32%)	516 (33%)		486 (31%)	509 (33%)	
Over 3.5	2274 (42%)	437 (40%)		474 (45%)	431 (40%)	
BMI			<.001			.6
<25	2372 (32%)	353 (25%)		345 (24%)	353 (26%)	
25≤/<30	2569 (35%)	505 (30%)		532 (33%)	498 (30%)	
≥30	2455 (33%)	688 (44%)		643 (43%)	669 (44%)	
Smoking			<.001			.9
Nonsmoker	4309 (59%)	676 (43%)		651 (42%)	667 (43%)	
Former smoker	1586 (22%)	534 (35%)		529 (36%)	524 (35%)	
Current smoker	1501 (19%)	336 (22%)		340 (22%)	329 (22%)	
Alcohol consumption			<.001			.6
Yes	5470 (79%)	1031 (71%)		1040 (75%)	1021 (71%)	
No	1926 (21%)	515 (29%)		480 (25%)	499 (29%)	
Physical activity status						
Vigorous			.005			.8
No	5862 (78%)	1304 (82%)		1276 (83%)	1281 (82%)	
Yes	1534 (22%)	242 (18%)		244 (17%)	239 (18%)	
Moderate			.037			.8
No	4512 (57%)	1003 (61%)		977 (58%)	984 (61%)	
Yes	2884 (43%)	543 (39%)		543 (42%)	536 (39%)	
Hypertension			<.001			>.9
No	5306 (75%)	651 (47%)		648 (45%)	651 (48%)	
Yes	2090 (25%)	895 (53%)		872 (55%)	869 (52%)	
Diabetes			<.001			.6
No	6706 (93%)	1216 (84%)		1215 (85%)	1199 (84%)	
Yes	690 (6.6%)	330 (16%)		305 (15%)	321 (16%)	
VAI (continuous)	1.34 (0.83, 2.27)	1.65 (1.05, 2.78)	<.001	1.62 (0.98, 2.64)	1.65 (1.05, 2.77)	.3
VAI (categorical)			<.001			.4
Q1	1929 (27%)	244 (16%)		397 (27%)	348 (23%)	
Q2	1800 (25%)	361 (25%)		374 (24%)	388 (26%)	
Q3	1858 (25%)	434 (27%)		379 (26%)	382 (24%)	
Q4	1809 (24%)	507 (32%)		370 (24%)	402 (26%)	

PIR = household income to poverty ratio, PSM = propensity score matching, VAI = visceral adiposity index.

* Chi-squared test with Rao and Scott second-order correction; Wilcoxon rank-sum test for complex survey samples.

### 3.2. VAI and RA risk relationships

**Figure 1.**
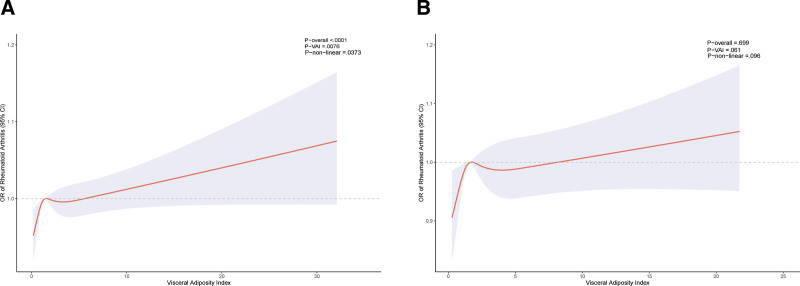
Restricted cubic spline for visceral adiposity index and the OR of rheumatoid arthritis before propensity score matching (A); restricted cubic spline for visceral adiposity index and the OR of rheumatoid arthritis after propensity score matching (B). CI = confidence interval. OR = odds ratio, VAI = visceral adiposity index.

To explore the association, a weighted multivariate logistic regression was conducted, and the results are summarized in Table 2. The model adjusting for all covariates (model 3) showed no significant relationship between continuous VAI and RA risk (*P* > .05). However, categorical VAI was significantly related to RA risk. Model 3 indicated a 3% increased RA risk for participants in the Q4 VAI group compared with those in the Q1 group (*P* = .015). The Q2 group also yielded a similar result. As illustrated in Figure 1A, the RCS analysis confirmed a strong nonlinear relationship between continuous VAI and the odds ratio of RA (*P*-nonlinear = .037, *P*-overall < .0001, *P*-VAI = .0076). Sensitivity analysis demonstrated that a higher VAI was positively associated with RA risk, a finding consistent with the results of the weighted logistic regression.

**Table 2:** Weighted multivariable logistic regression for the association between the VAI and rheumatoid arthritis risk.

	OR (95% CI), *P* value		
	Model 1	Model 2	Model 3
Continuous			
VAI	1.01 (1.00–1.01), <.001	1.00 (1.00–101), .019	1.00 (1.00–1.01), .11
Categories			
Q1	Reference	Reference	Reference
Q2	1.06 (1.03–1.08), <.001	1.03 (1.01–1.05), .012	1.02 (1.00–1.05), .028
Q3	1.07 (1.04–1.09), <.001	1.02 (1.00–1.05), .037	1.02 (1.00–1.04), .14
Q4	1.10 (1.08–1.12), <.001	1.04 (1.02–1.06), <.001	1.03 (1.01–1.05), .015

Model 1 was adjusted for no covariates.

Model 2 was adjusted for age, sex, race, education level, marital status, ratio of family income to poverty, and body mass index.

Model 3 was adjusted for covariates in model 2 + smoking, alcohol consumption, physical activity status, hypertension, and diabetes.

CI = confidence interval, OR = odds ratio, VAI = visceral adiposity index.

### 3.3. Subgroup analysis

A subgroup analysis was conducted to assess the stability of the VAI-RA relationship across different stratifications. Results revealed that the 20- to 39-year-old, male, current smoker, alcohol consumption, non-hypertension, and non-diabetes subgroups exhibited a consistent relationship between VAI and RA risk (*P* < .05, Table 3). However, the relationship may be influenced by age and gender variables (*P* value for interaction < .05). Notably, the alcohol consumption group exhibited a higher RA risk compared with the non-alcohol consumption group. A positive relationship between VAI and RA risk was observed in each quartile level of the alcohol consumption group (Q2: 1.03, Q3: 1.02, Q4: 1.04).

**Table 3:** Subgroup analysis for the relationship between VAI and rheumatoid arthritis risk before PSM.

Subgroups	OR (95% CI), *P* value					*P* value for interaction*	
	Q1	Q2	Q3		Q4		
Age (yr)	Reference						.02
20–39	Reference	1.02 (0.98–1.02), >.90		1.03 (1.01–1.05), .01	1.02 (0.99–1.04), .20		
40–59	Reference	1.05 (1.00–1.10), .06		1.01 (0.96–1.05), .80	1.04 (1.00–1.08), .068		
Over 60	Reference	1.05 (0.99–1.12), .13		1.03 (0.96–1.10), .40	1.04 (0.95–1.13), .40		
Sex							.03
Male	Reference	1.03 (1.00–1.06), .06		1.04 (1.01–1.07), .02	1.02 (0.99–1.05), .30		
Female	Reference	1.02 (0.99–1.05), .20		1.00 (0.96–1.04), .90	1.03 (1.00–1.07), .06		
Smoking							>.05
Nonsmoker	Reference	1.02 (0.99–1.04), .20		1.01 (0.99–1.04), .30	1.01 (0.98–1.04), .40		
Former smoker	Reference	1.01 (0.97–1.07), .60		1.02 (0.97–1.07), .50	1.05 (0.99–1.11), .10		
Current smoker	Reference	1.07 (1.01–1.13), .02		1.05 (0.99–1.11), .13	1.07 (1.00–1.15), .06		
Alcohol consumption							>.05
Yes	Reference	1.03 (1.00–1.05), .04		1.02 (1.00–1.04), .07	1.04 (1.01–1.07), .004		
No	Reference	1.03 (0.97–1.10), .30		1.01 (0.96–1.08), .60	1.01 (0.95–1.06), .80		
Hypertension							>.05
Yes	Reference	1.05 (0.99–1.12), .09		1.04 (0.98–1.11), .2	1.04 (0.98–1.10), .2		
No	Reference	1.02 (1.00–1.04), .07		1.01 (0.99–1.04), .30	1.04 (1.01–1.06), .006		
Diabetes							>.05
Yes	Reference	0.94 (0.82–1.06), .30		0.93 (0.83–1.05), .20	0.94 (0.86–1.04), .20		
No	Reference	1.03 (1.01–1.05), .007		1.03 (1.00–1.05), .03	1.04 (1.01–1.06), .006		

CI = confidence interval, OR = odds ratio, PSM = propensity score matching, VAI = visceral adiposity index.

* Interaction analysis between the selected subgroup and model 3.

### 3.4. PSM analysis

After performing a 1:1 PSM, 1520 RA participants and 1520 non-RA participants were included (Table 1). Notably, the RA and non-RA groups showed no statistically significant differences in continuous VAI and categorical VAI. Figure S2, illustrates the data distribution before and after PSM.

As shown in Table 4, continuous VAI and RA risk exhibited no significant correlations in the weighted multivariable logistic regression analysis (*P* > .05). Similarly, Figure 1B, based on RCS analyses, indicated that continuous VAI was not significantly associated with RA risk in the fully adjusted model (*P*-nonlinear > .05, *P*-overall > .05). However, in model 3, it was found that Q2 VAI was related to RA risk, significantly higher than Q1 VAI (*P* = .03). The sensitivity analysis yielded a *P* value of .052, indicating a potential association between VAI and RA risk. Furthermore, the relationship between categorical VAI and RA risk in each subgroup was summarized in Table 5. The results indicated that categorical VAI was associated with an increased RA risk in the alcohol consumption, non-hypertension, and non-diabetes subgroups. Particularly in the alcohol consumption group, Q4 VAI had a higher RA risk than Q1 VAI (*P* = .045). In contrast to the pre-PSM results, no significant interaction effects between subgroup variables and the VAI-RA risk were found post-PSM (*P* value for interaction > .05).

**Table 4:** Weighted multivariable logistic regression for the association between the VAI and rheumatoid arthritis risk after PSM.

	OR (95% CI), *P* value		
	Model 1	Model 2	Model 3
Continuous			
VAI	1.00 (1.00–1.01), .09	1.00 (1.00–1.01), .20	1.00 (1.00–1.01), .12
Categories			
Q1	Reference	Reference	Reference
Q2	1.07 (1.00–1.14), .04	1.07 (1.00–1.14), .05	1.07 (1.01–1.15), .03
Q3	1.03 (0.96–1.10), .40	1.03 (0.96–1.10), .40	1.04 (0.97–1.11), .30
Q4	1.06 (0.99–1.14), .09	1.06 (0.98–1.14), .13	1.07 (0.99–1.16), .07

Model 1 was adjusted for no covariates.

Model 2 was adjusted for age, sex, race, education level, marital status, ratio of family income to poverty, and body mass index.

Model 3 was adjusted for covariates in model 2 + smoking, physical activity status, alcohol consumption, hypertension, and diabetes.

CI = confidence interval, OR = odds ratio, PSM = propensity score matching, VAI = visceral adiposity index.

**Table 5:** Subgroup analysis for the relationship between VAI and rheumatoid arthritis risk after PSM.

Subgroups	OR (95% CI), *P* value					*P* value for interaction*
	Q1	Q2	Q3	Q4		
Age (yr)						>.05
20–39	Reference	1.08 (0.91–1.30), .40	1.15 (0.93–1.42), .90		1.12 (0.92–1.35), .30	
40–59	Reference	1.06 (0.94–1.19), .30	0.99 (0.87–1.12), .80		1.05 (0.92–1.20), .50	
Over 60	Reference	1.06 (0.97–1.15), .20	1.02 (0.92–1.13), .70		1.05 (0.95–1.17), .30	
Sex						>.05
Male	Reference	1.09 (1.00–1.19), .06	1.08 (0.98–1.20), .12		1.06 (0.95–1.18), .30	
Female	Reference	1.05 (0.95–1.15), .40	0.99 (0.91–1.09), .80		1.05 (0.95–1.16), .30	
Smoking						>.05
Nonsmoker	Reference	1.05 (0.96–1.14), .30	1.04 (0.94–1.14), .50		1.05 (0.96–1.16), .30	
Former smoker	Reference	1.07 (0.96–1.18), .20	1.04 (0.93–1.16), .50		1.09 (0.97–1.22), .14	
Current smoker	Reference	1.07 (0.91–1.25), .40	0.96 (0.82–1.13), .60		1.05 (0.86–1.27), .60	
Alcohol consumption						>.05
Yes	Reference	1.10 (1.03–1.18), .007	1.02 (0.95–1.10), .60		1.10 (1.00–1.20), .045	
No	Reference	0.99 (0.87–1.14), .90	1.05 (0.91–1.21), .50		0.99 (0.86–1.13), .80	
Hypertension						>.05
Yes	Reference	1.03 (0.93–1.14), .50	1.01 (0.93–1.11), .80		1.06 (0.96–1.16), .30	
No	Reference	1.12 (1.03–1.22), .008	1.05 (0.95–1.16), .30		1.08 (0.95–1.22), .20	
Diabetes						>.05
Yes	Reference	0.93 (0.77–1.13), .50	0.95 (0.79–1.14), .60		1.00 (0.86–1.16), >.90	
No	Reference	1.10 (1.02–1.18), .01	1.04 (0.96–1.13), .30		1.08 (0.99–1.17), .10	

CI = confidence interval, OR = odds ratio, PSM = propensity score matching, VAI = visceral adiposity index.

* Interaction analysis in model 3.

## 4. Discussion

This study involved 8942 participants, comprising 1546 RA patients and 7396 controls. Weighted multiple logistic regression analysis, adjusting for marital status, educational level, age, sex, PIR, and BMI, revealed a significant association between VAI (both continuous and categorical) and an increased risk of RA. RCS analysis further supported a relationship between continuous VAI and RA risk (*P*-VAI = .0078). However, in the all-adjusted weighted multiple logistic regression analysis, only the Q2/4 group exhibited a significantly higher RA risk compared with the Q1 group. Additional covariates, including hypertension, physical activity, smoking, diabetes, and alcohol consumption, may play crucial roles in influencing RA risk. Conducting a subgroup analysis, we found that the Q2/4 group had a higher RA risk, particularly within the alcohol consumption subgroup. Recognizing potential selection bias, we performed a 1:1 PSM, resulting in 1520 RA patients and 1520 control participants. In the all-adjusted weighted multiple logistic regression analysis post-PSM, only the Q2 group demonstrated a higher RA risk than the Q1 group (*P* = .03). Moreover, RCS analysis indicated no significant relationship between continuous VAI and RA risk (*P*-VAI = .061). The subgroup analysis post-PSM yielded consistent results with those before PSM, showing a 10% higher RA risk in alcohol consumption participants within the Q2/4 group compared to the Q1 group. Overall, our study suggests that populations with high VAI levels, particularly those with alcohol consumption, non-hypertension, and non-diabetes, exhibit an increased risk of RA. The reliability of our results is supported by the consistent use of similar research methods in previous studies. While our study identified a statistically significant association between the VAI and RA risk, the observed effect sizes were modest, with ORs approximating 1.01 to 1.05. These results suggest that visceral adiposity is not a strong, direct risk factor at the individual level but rather a component within the disease’s broader, complex etiology. Consequently, the VAI is not a definitive diagnostic tool for individual risk prediction. It may, however, serve as an easily obtainable clinical indicator that prompts heightened vigilance and encourages healthier lifestyle choices in patients with high visceral adiposity.

This study used the VAI as an obesity-related measure to indirectly assess the association between obesity and RA risk. Previous studies have linked obesity to an increased risk of RA. In a case-control study, Dar et al examined 11,406 RA patients and 54,701 controls, revealing a higher proportion of obesity in the RA group.^[13]^ Multivariate regression analysis further confirmed the association between obesity and RA, with potential mechanisms involving activated circulating inflammatory biomarkers, elevated sex hormone levels, and vitamin D deficiency. Similarly, Linauskas et al conducted a Danish cohort study, establishing a positive correlation between body fat and RA risk in the female population.^[14]^ Adipose tissue, through the secretion of pro-inflammatory agents, is implicated in promoting systemic inflammation. Meta-analysis, considered the gold standard in evidence-based medicine, reinforces the relationship between obesity and RA. A meta-analysis by Ohno et al encompassing 10 cohort studies suggests that a 5 kg/m^2^ increase in BMI is associated with an 11% relative risk of RA.^[15]^ Furthermore, Mendelian randomization studies support a causal effect of obesity on RA. In a Mendelian randomization study, Karlsson et al demonstrated that a high BMI may be linked to an increased risk of RA.^[16]^ Similarly, a 2-sample Mendelian randomization study by Bae et al demonstrated a causal link between increased BMI and heightened RA risk.^[17]^ Notably, obesity-related single-nucleotide polymorphisms were associated with hypothalamic signal transduction, suggesting a potential role of hypothalamic inflammation in RA development. Interestingly, bariatric surgery emerges as a potential modulator of RA prognosis. Lin et al reported a reduction in the in-hospital mortality rate for RA patients undergoing bariatric surgery.^[18]^ A systematic review by Miladi et al further indicated that bariatric surgery could mitigate RA development and related mortality in obese individuals.^[19]^ Collectively, our study, along with previous research, suggests that obesity significantly influences the risk of RA.

RA patients often exhibit concurrent muscle loss and increased body fat. Despite a constant BMI, there is a significant increase in body fat among these patients. The association between type 2 diabetes, metabolic syndrome, and insulin resistance with VAI has been well established. In a cross-sectional study based on the NHANES, Jiang et al identified an important link between insulin resistance and VAI.^[20]^ Similarly, Cárdenas et al, in a retrospective study involving 1372 individuals, reported a remarkably high predictive value of 93.2% for VAI in determining metabolic syndrome.^[21]^ A cohort meta-analysis by Jayedi et al further confirmed a positive link between VAI and type 2 diabetes risk.^[22]^ Notably, previous research has highlighted associations between these metabolic conditions and RA.^[23,24]^ Although these studies do not directly explore the relationship between VAI and RA, their findings suggest a potential link between visceral adiposity and the risk of RA. Beyond WC and BMI, other obesity-related indicators have been reported to correlate with RA risk. Wang et al conducted a study based on NHANES, revealing a positive correlation between a weight-adjusted waist index and the risk of RA.^[25]^ The authors suggest that an imbalance in obesity-related intestinal flora and systemic inflammation may underlie the pathogenesis of RA. After employing PSM, only the Q2 group exhibited a higher RA risk than the Q1 group in the alcohol consumption, non-hypertension, and non-diabetes subgroups. In addition, subgroup analysis did not suggest the presence of interactions. Therefore, our results indicate that visceral obesity may be associated with RA risk specifically within a defined population.

This is the first study to demonstrate a correlation between elevated VAI and an increased risk of RA. Furthermore, the stability of the results post-PSM analysis enhances the robustness of our findings. However, this study also has some unavoidable limitations. First, the results from this analysis apply to the general US population, as the NHANES serves as a representative survey of this demographic. Second, due to the cross-sectional nature of the study, establishing a causal link between RA risk and VAI is precluded. Third, while our study identifies the relationship, further investigations are imperative to elucidate the underlying mechanisms. Fourth, despite the widespread recognition of BMI, it remains an imperfect body index. VAI, like other newly proposed physical indicators, cannot entirely replace BMI. Last, the study does not account for potential covariates that might influence the results due to incomplete data. The limitations of our study highlight priorities for future research. Longitudinal studies are required to establish the temporal relationship between visceral adiposity and RA onset, thereby addressing the constraints of the cross-sectional design. Employing clinically verified diagnoses rather than self-reported ones, and supplementing the VAI with imaging-based fat measurements, would enhance measurement accuracy. Furthermore, our findings underscore the need to investigate the specific inflammatory pathways that connect visceral fat to RA. Finally, interventional trials are necessary to determine whether reducing visceral adiposity can effectively lower the risk of RA.

## 5. Conclusion

Among US adults, an elevated VAI is associated with an increased risk of RA, particularly in populations characterized by alcohol consumption, non-hypertension, and non-diabetes. Notably, alcohol consumers with a high VAI face an even higher RA risk. While these findings suggest an association between visceral adiposity and RA development, the cross-sectional design precludes causal inference. Prospective studies are required to confirm these observations and to investigate the underlying mechanisms.

## Acknowledgments

We thank the Home for Researchers editorial team (www.home-for-researchers.com) for language editing service.

## Author contributions

**Software:** Dianbo Yu.

**Data curation:** Wei Huang.

**Visualization:** Junnian Yang.

**Writing – review & editing:** Jun Yao.

**Writing – original draft:** Shengcong Guo.



**Figure:**