# Supplementary figures and images for: Association between the visceral adiposity index and rheumatoid arthritis: A cross-sectional study based on the NHANES 2007 to 2016
Source: Medicine (Baltimore). 2026 May 22;105(21):e48744. doi: 10.1097/MD.0000000000048744 (PMC13201013; doi:10.1097/MD.0000000000048744)

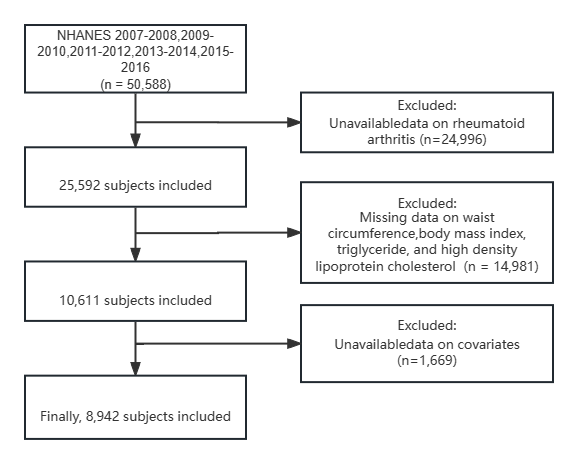

Supplement: Supplementary file 1 [file medi-105-e48744-s001.png]
